# Do pregnant people have opportunities to participate in clinical trials? an exploratory survey of NIHR HTA-funded trialists

**DOI:** 10.1186/s13063-025-08949-w

**Published:** 2025-07-04

**Authors:** Rebekah Burrow, Lisa Hinton, Mike Clarke

**Affiliations:** 1 Nuffield Department of Primary Care Health Sciences, University of Oxford, Oxford, UK; 2 Queen’s University Belfast, Belfast, Northern Ireland

**Keywords:** Clinical trials, Inclusion, Pregnancy, Survey, HTA

## Abstract

**Background:**

Pregnant people are often excluded from clinical trials, primarily due to safety concerns. However, exclusion causes population-level harms as well as sometimes providing individual protection. Harms caused to pregnant people by exclusion from clinical trials have been clearly evidenced and highlighted during the COVID pandemic. The National Institute for Health and Care Research (NIHR) has since provided guidance on improving inclusion of under-served groups, which includes pregnant people, in clinical research. Appropriate inclusion and active facilitation to participate are required to provide equitable evidence-based healthcare during pregnancy and to comply with ethical principles for research.

**Methods:**

We carried out an exploratory, online, cross-sectional survey of trialists to assess whether, why, and how pregnant people are included or excluded from clinical trials funded by the NIHR Health Technology Assessment (HTA) Programme, with awards starting in 2022–2023. Trialists were the respondents, with trials the primary focus of this survey. Invitations were sent to trialists between October 2023 and March 2024. Summary statistics were calculated to describe the characteristics of the trials and respondents, to describe eligibility of pregnant people, reasons for this, and how this eligibility is documented and implemented.

**Results:**

We identified 120 trials of which 88 were eligible for this survey. Responses were received for 81 trials. Pregnant people are excluded from 34 of these 81 trials. Pregnant people are eligible for inclusion in 40 of the 81 trials, including four which partially exclude people during pregnancy. Eligibility is unclear for seven trials. Exclusions are mostly for safety reasons. Sponsors and regulatory authorities are unnecessary barriers to inclusion in some trials. Eight trials of 40 trials make explicit or deliberate attempts to include people during pregnancy.

**Conclusions:**

A minority of the 120 trials include people during pregnancy. Most trials for which pregnant people are eligible do not report explicitly including people during pregnancy or facilitating their inclusion. A small number of trials, different in setting, clinical area, and intervention type, are intentionally designed and conducted in a way that include people during pregnancy. There are clear opportunities to improve the inclusion of pregnant people in clinical trials in the NIHR HTA Programme.

**Supplementary Information:**

The online version contains supplementary material available at 10.1186/s13063-025-08949-w.

## Background

Pregnant people are often excluded from clinical trials primarily due to safety concerns for the foetus in pharmaceutical trials [1–5]. However, although exclusion from clinical trials sometimes provides immediate protection to individuals who are not included, it also causes subsequent population-level harms by creating an evidence gap that leads to withholding or withdrawing of safe and effective treatment in healthcare [6, 7]. Harms caused to pregnant people by exclusion from clinical trials have been clearly evidenced and highlighted during the COVID pandemic [6, 8]. This runs counter to Principle 13 of The Declaration of Helsinki which states “Groups that are underrepresented in medical research should be provided appropriate access to participation in research” [9]. Pregnant people report altruistic and personal reasons for their willingness to participate in clinical research [10].

There are various efforts to fill evidence gaps for people during pregnancy through inclusion in clinical trials and epidemiological studies; a number of initiatives have been formed to advance work in this space [11–14]. In 2022, the National Institute for Health and Care Research (NIHR) strategy included a theme “Widen access and participation for greater diversity and inclusion” [15]. The NIHR INCLUDE project has provided guidance on improving inclusion of under-served groups in clinical research [16]. Pregnant people, women of child-bearing age, and women are all under-served groups. Appropriate inclusion and active facilitation to participate are required to provide equitable evidence-based healthcare during pregnancy and to comply with ethical principles for research [12, 16–19].

The NIHR is the British government’s largest funder of health and care research and its HTA Programme is the largest funding programme of clinical trials. HTA-funded clinical trials evaluate clinical and cost-effectiveness of interventions used in the NHS for the treatment, prevention, or diagnosis of disease, so are likely to be later phase trials; later phase trials are more likely to include pregnant women. These interventions include but are not limited to, procedures, pharmacological, devices, therapies, and diagnostics. This single funding stream may be the most likely to have the highest rates of inclusion of pregnant people. This study fills a gap by providing data on inclusion and participation of people during pregnancy in NIHR HTA clinical trials and quantifying the rates of inclusion of pregnant people in a sample of clinical trials that is likely to be more inclusive than many others.

We incorporated patient and public involvement (PPI) to ensure that this study of research methodology remained relevant to people with experience of pregnancy.

Previous research has found that the eligibility of pregnant people for clinical trials was not clear for a large minority of trial registrations or published results [1, 8]. Therefore, we carried out an exploratory survey of trialists working on NIHR HTA Programme-funded clinical trials. We targeted trialists working on trials with funding awards beginning in 2022–2023 where participants could feasibly include pregnant people. We aimed to assess whether, why, and how pregnant people are included or excluded from these clinical trials.

This survey provides a snapshot of the current rates of inclusion of people during pregnancy from the point of view of trialists and as such creates a baseline from which change can be measured. The survey also explores reasons for exclusion, and how this eligibility is documented and implemented. For example, this might be through a statement in the protocol that people are eligible during pregnancy, or exclusion through requiring a negative pregnancy test. These data can be used to identify barriers to inclusion.

## Methods

### Survey design

We used an exploratory, online, cross-sectional survey, available in the supplementary material. We developed two similar versions of the survey, one for trials which explicitly excluded people during pregnancy, and another for trials for which eligibility was not clear. Initial questions were used to identify the trial and the post of the respondent. Subsequent questions asked about the eligibility of pregnant people to participate, and based on their answer, allowed respondents to choose how to answer further questions about how and why they were eligible or not. The survey had 9–10 pages with a total of 4–7 questions, depending on the answers given. We used multiple-choice answers followed by a space for free-text to maximise broad and informative responses while minimising effort and time required by respondents.

### Survey testing

The email invitation and survey were tested by seven researchers, five of whom had experience of working on clinical trials; the email invitation and survey were refined based on their feedback. The email and survey were again piloted through invitations to 11 potential respondents for a random sample of six trials; these responses were included in the final results and no further refinements were made.

### Study population

The study target population was:

- Trialists working on NIHR HTA-funded clinical trials
- For clinical trials identified through the NIHR Funding Awards Website (https://fundingawards.nihr.ac.uk/)
- With a funding award start date between 1 January 2022 and 31 December 2023
- With a trial population that could potentially include people during pregnancy (for example, excluding trials of people aged only 60 or above, or of only people with male reproductive organs)

### Survey administration

For trials meeting these criteria, we sent invitations to those listed as “chief investigator” or “joint lead applicant”, with a reminder email after two or more weeks. In the event of no response, an invitation was sent to the trial email address, or, if none could be found, to a trial manager. Again, a reminder was sent after two or more weeks. When any response was received from a trial, we stopped sending invitations to all potential respondents for that trial. When all eligible trials had been invited and at least 2 weeks allowed for a response to the last reminder email, the survey was closed. In order to maximise information collection and minimise annoyance to respondents, we considered either a formal response to the survey, or an email reply with any relevant information as a response. Only complete surveys were saved.

The survey was administered in JISC Online Surveys v2 and open for responses from 5 October 2023 to 31 March 2024. Multiple entries were expected from some individuals who worked on more than one trial, and were all included in the analysis. Multiple entries for a trial from the same or different individuals were identified from the trial and job selected, and were collated and included in the analysis.

### Analysis

Trials, and information about interventions, settings, and clinical conditions, were identified through information available on the NIHR Awards webpages. All other information was provided by respondents; the survey is provided in supplementary file 1.

Trialists were the respondents and they provided information about trials. The trials are the primary focus of this survey; the results describe the trials unless it is explicitly stated that that information refers to respondents.

Unique site visitors and view rate were not determined because no data was collected to enable this. Respondent progress was determined by drop-out rates per page. Representativeness of the target population was assessed by comparing the characteristics of trials for which we did and did not receive responses. Non-response rate and potential reasons were considered.

Free-text answers were coded by one person who manually assigned a value or values to each response.

When information in email responses could be clearly linked to a specific survey question, the information was analysed in the same way as a free-text answer to that question in a survey response. When information in emails was not clear or did not answer a specific survey question, it was not included in the analysis.

Summary statistics were calculated to describe the characteristics of the trials and respondents, to describe eligibility of pregnant people, reasons for this, and how this eligibility is documented and implemented. No statistical correction methods were used and no sensitivity analysis was conducted. Excel was used for data analysis.

### Patient and public involvement

We recruited PPI contributors through a community-based family centre, a primary school, and a pregnancy yoga group. We carried out individual interviews with five contributors and asked about their experiences of pregnancy, including their access to treatments, information about treatments, and participation in research. We also asked about their opinions of, and interest in, research to increase inclusion in research during pregnancy. We asked one contributor to review our results and the way that we had reported them. We have reported PPI following the GRIPP2 short form reporting checklist [20].

## Results

### Respondent characteristics

**Fig. 1:**
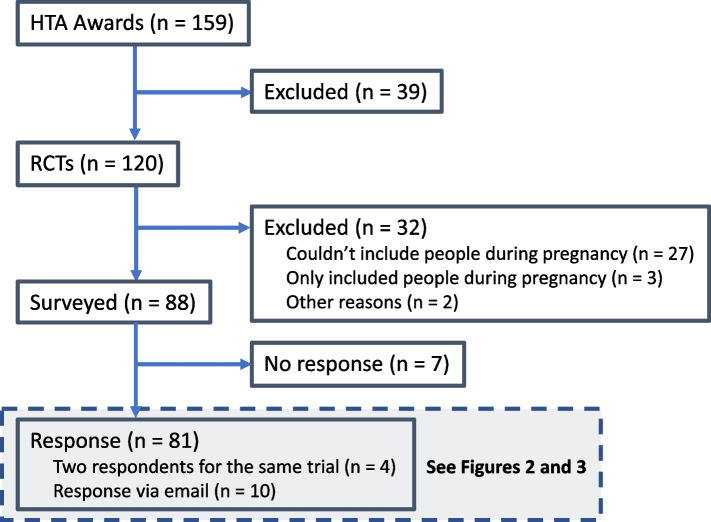
Flow diagram: eligibility of trials for inclusion in this survey

One hundred and twenty trials were identified with awards starting 2022–2023, of which 27 cannot include people during pregnancy (for example, a trial including only children) and three only include people during pregnancy (for example, a trial in gestational diabetes) (Fig. 1), and two were excluded for other reasons. Eighty-eight eligible trials were invited to participate through invitations to 130 chief investigators or joint lead applicants, and invitations to 20 trials teams or trial managers. Responses were received for 81 trials (92% of the target population) through 74 survey responses (including two responses from four trials) and 11 email responses. Seventy responses were from a senior member of the trial team such as the chief investigator. Fifteen were from members of the trial team responsible for conduct, such as a trial manager.

### Non-response

Reasons provided by respondents for initial no response, or partial response via email rather than through the survey were:

- Respondents’ firewalls blocking access to the survey
- That their trial could not be captured in the response options

Respondent progress through the survey showed that 95% of survey drop-outs occurred on the two pages of the participant information sheet and privacy notice.

### Main findings

Characteristics of the surveyed trials derived from the NIHR HTA Awards webpages are summarised in Table 1. Interventions under study are mostly pharmacological, procedures (most of which are surgical), and devices. Most trials take place in a hospital setting. Interventions are focused on patients with a large range of clinical conditions. The trials for which a response was not received are similar in these characteristics to trials for which a response was received.

**Table 1:** Characteristics of trials in the target population

	**Respondent**	**Non-respondent**
*n* =	81	7
Intervention type		
Pharmacological	27 (33.3%)	4 (57.1%)
Procedure	21 (25.9%)	0
Device	12 (14.8%)	0
Biologic	4 (4.9%)	0
Support	4 (4.9%)	0
Device and procedure	2 (2.5%)	0
Education	2 (2.5%)	0
Procedure and support	2 (2.5%)	0
Therapy	2 (2.5%)	0
Other	5 (6.2%)	3 (42.9%)
Setting		
Community	9 (11.1%)	1 (14.3%)
Primary care	3 (3.7%)	2 (28.6%)
Community and hospital	1 (1.2%)	0
Primary and hospital	7 (8.6%)	0
Hospital	58 (71.6%)	3 (42.9%)
Unclear	3 (3.7%)	1 (14.3%)
Clinical condition		
Orthopaedic	12 (14.8%)	2 (28.6%)
Psychiatric	9 (11.1%)	2 (28.6%)
Cardiovascular	9 (11.1%)	2 (28.6%)
Oncology	8 (9.9%)	0
Infection	7 (8.6%)	1 (14.3%)
Respiratory	6 (7.4%)	0
Anaesthesiology	5 (6.2%)	0
Rheumatology	4 (4.9%)	0
Emergency care	4 (4.9%)	0
Endocrinology	3 (3.7%)	0
Neurology	3 (3.7%)	0
Haematology	3 (3.7%)	0
Dermatology	2 (2.5%)	0
Other	6 (7.4%)	0

Each trial is counted once in each section of the table.

Intervention: “Other” includes behavioural, care, device, dietary, pharmacological, education, procedure, support, therapy, and training, variously alone and/or in combination.

Setting: Hospital/secondary/tertiary settings were not differentiated because it was not possible to accurately assess and usefully tabulate settings in more detail based on the information available.

Clinical condition: “Other” includes intensive care, nephrology, occupational health, gastroenterology, ophthalmology, and audiology. When more than one clinical condition was relevant to a trial, one condition relevant to the condition based on which participants were included in the trial was selected (for example, a trial of post-operative antibiotics for a fracture was classed as “orthopaedic” rather than “infection”).

### Unclear eligibility

**Fig. 2:**
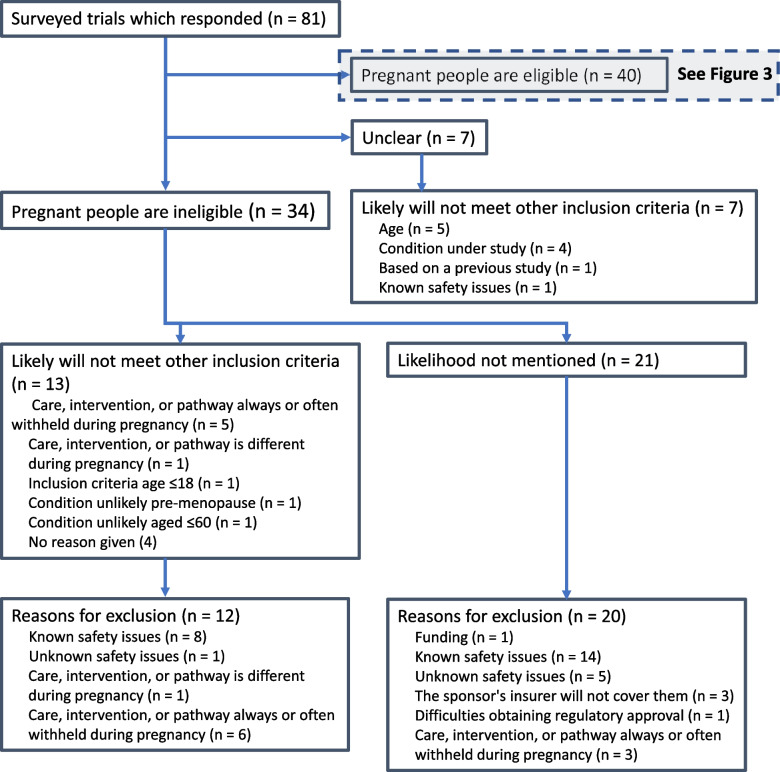
Flow diagram: ineligibility of people during pregnancy Legend: Not all trialists volunteered an assessment of whether pregnant people would be likely to meet other eligibility criteria. Trialists could give no reason or multiple reasons that pregnant people would not meet other inclusion criteria. Trialists could give no reason or multiple reasons for excluding people during pregnancy

For seven trials, it was unclear from the responses whether people were eligible during pregnancy, or not (Fig. 2). The majority of unclear responses were received via email. The respondents for these seven trials reported that it is unlikely that pregnant people would meet the inclusion criteria for reasons other than pregnancy—mainly due to age, or the condition under study.

### Ineligibility

Of the 81 trials, 34 responded that people are ineligible during pregnancy. Thirteen of these 34 stated that pregnant people are unlikely to meet other inclusion criteria. Of these 13, four trials did not provide an explanation of why pregnant people would not meet other inclusion criteria. Of the 13, three trials considered the age of participants, or the very low burden of disease on patients of an age compatible with pregnancy, to make pregnant participants unlikely (for example, a trial for a condition with highest incidence in over 75-year-olds). Six trials reported that an intervention can be delayed until after pregnancy, or that the care, intervention, or pathway would be different during pregnancy (for example, a trial of an intervention for diabetes would differ in usual care and setting from that during pregnancy). The reasons given for explicitly excluding people during pregnancy were similar for trials whether or not pregnant people were considered likely to meet other inclusion criteria. Twenty-five trials reported safety issues as the reason for ineligibility. Respondents included detail in free-text answers about why they are unable (or anticipate being unable) to include people during pregnancy in the absence of safety concerns. These included issues with sponsor insurance and regulatory authorities.

- Trial 5 “*This is a pragmatic trial and I didn’t want the pregnancy exclusion. We were behind schedule at this point and arguing [with an external party] seemed a potentially fruitless task we didn’t have time for – and the gain in terms of likely number of pregnant recruits was minimal*”
- Trial 57 “*There are no adverse effects expected should a participant become pregnant whilst on the trial but because they cannot be insured, they must be withdrawn (even if they are on the standard care arm).*”

Twenty-eight trials identified at least one way that ineligibility due to pregnancy is documented or implemented, with six trials describing the use of pregnancy tests or contraception.

### Eligibility

**Fig. 3:**
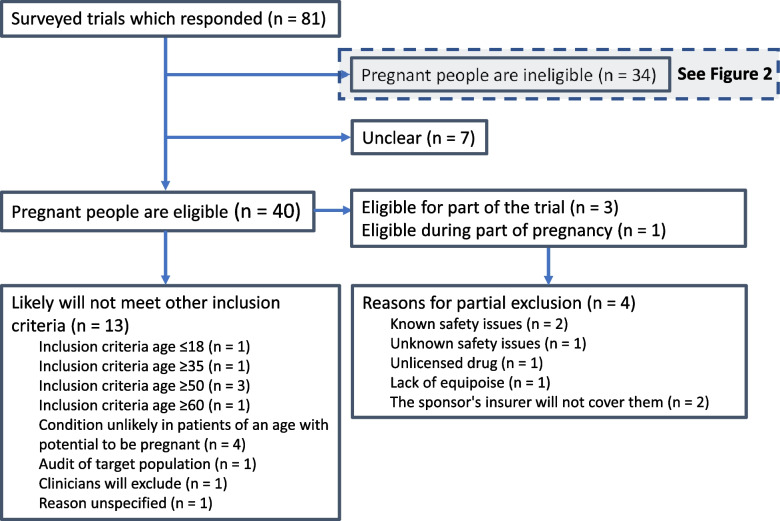
Flow diagram: eligibility of people during pregnancy. Legend: Not all trialists volunteered an assessment of whether pregnant people would be likely to meet other eligibility criteria. Trialists could give no reason or multiple reasons that pregnant people would not meet other inclusion criteria. Trialists could give no reason or multiple reasons for excluding people during pregnancy

Forty trials reported that people are eligible during pregnancy (Fig. 3). This included four trials which partially exclude people during pregnancy but purposefully include people during pregnancy in as much of the trial as possible (for example, through inclusion in some arms of a platform trial). Safety is the main reason that pregnant people are not being included in the whole trial, and lack of equipoise is the main reason that pregnant people are not being included at all stages of pregnancy.

Of the 36 trials in which people are fully eligible during pregnancy, 13 reported that it is likely no pregnant people will meet the inclusion criteria. This judgement was mostly based on the prevalence of conditions by age and age-based eligibility criteria. These assumptions seemed mostly reasonable, although trialists may be underestimating the prevalence of multimorbidity in pregnant women (which is unknown) and polypharmacy (which is between 5 and 62% [21]).

Twenty-two trials of the 36 trials identified at least one way that eligibility in spite of pregnancy is documented or implemented.

Of all 40 trials that reported that people are eligible during pregnancy, eight described deliberate attempts to include people during pregnancy, including negotiating with the sponsor, checking insurance policies, tailoring interventions to suit their needs, and excluding active participation of patients in specific trial arms post-randomisation.

- Trial 29 “*participants are advised that if randomized to the intervention group the intervention is tailored to suit their needs and this tailoring would enable them to participate.*”
- Trial 59 “*they are not excluded. We faced this issue in a different trial… recently and found a solution to ensure pregnant people were not excluded, which will apply to this trial too. It wasn’t simple to resolve though, including needing to check the trials insurance policy and clarify processes for the interventions.*”
- Trial 58 “*Pregnant patients can be included but are excluded from some specific interventions… We state which interventions are and are not suitable for pregnancy*”

Twelve of the 36 trials in which people are fully eligible during pregnancy reported that, although pregnant people are eligible for inclusion, this is not explicit in the trial documentation and there is no targeted action to include them specifically.

Respondents volunteered information about why they tried to include people during pregnancy. They gave a wide variety of reasons including pragmatic design, the balance of likely harms and benefits in pregnancy, and the absence of a reason not to include them. Additional quotes are available in the supplementary material.

- Trial 13 “*We are not excluding pregnant people as [condition] is more harmful than [intervention being tested].*”
- Trial 20 “*Pregnant women get [condition] and are able to have [intervention being tested]*”
- Trial 70 “*Pregnant patients included as routine and also an appendix to expand any particular considerations*”

Some trialists made statements that are inaccurate, for example, that [pregnant] women do not undergo short-stay surgical procedures [22]. Assumptions were also made about age and the likelihood of pregnancy—for example, that pregnancies were very unlikely to occur in people aged 12–18, or that there would be a very low number of pregnant people aged 35 and over and, again, evidence does not support this generally [23]. With around 825,000 known conceptions per year in the UK, an increasing proportion of which are in older women compared to 2011, trialists who do not routinely work with pregnant people may be underestimating the prevalence of pregnancy in their patient populations [23].

The feedback from our five PPI contributors reinforced our interest in this project. We made changes to the way we described these results in response to the additional feedback from one PPI contributor.

## Discussion

Trials funded through the NIHR HTA Programme that started in 2022–2023 include a range of types of interventions, settings, and clinical conditions.

Of 120 trials, three include only pregnant people; as the NIHR and UK government’s 2022 strategies prioritising research in women’s health research take effect, we might see an increase in trials funded by the NIHR and others, focusing on pregnancy [24, 25].

Respondents reported that pregnant people were eligible for inclusion in 40 trials, of which eight are intentionally designed and conducted in a way that includes people during pregnancy. As trialists’ practices become more inclusive through the use of new guidance and tools, they may actively include more under-served groups generally, which could increase inclusion of pregnant people specifically [16, 26].

The majority of the 120 NIHR HTA-funded trials do not include people during pregnancy, mostly due to the conditions under study in these trials meaning that pregnant people cannot plausibly be included. A minority of trials did not include people during pregnancy because the care, intervention, or pathway during pregnancy is different to that in the trial.

Many trials cited safety concerns related to the intervention(s) under study. These are challenging trials in which to include people during pregnancy, and as others have advised, the potential harm: benefit ratio for pregnant people needs to be considered, rather than presence of potential harms alone [7]. Respondents reported that a small number of trials did not include people during pregnancy in spite of clinical assessment that it would be safe to do so. This was due to decisions made by, or pressure applied by, sponsors and/or regulatory authorities. Increasing prioritisation of inclusivity of trials may help encourage organisations to support more inclusive research.

Some trialists working on trials in a variety of settings, clinical areas, and interventions felt it was obvious, easy, or necessary to include people during pregnancy, which could indicate that trialists would be receptive to specific support for inclusion of people during pregnancy; this could be an area for future research to explore motivations of trialists, identify facilitators of inclusion, and describe exemplars.

Many trialists made assumptions about what sort of participants were likely to be pregnant, mostly based on age and condition. Although some may often be correct in these assumptions, they are not always correct and, collectively, these trials are likely to encounter some pregnant participants, an experience one trial of post-surgical care reported. Tackling these assumptions could be one way to increase inclusion in trials during pregnancy.

A small number of trials in this sample are intentionally designed and conducted in a way that include people during pregnancy. These include some trials that may have safety issues, of interventions that some consider incompatible with pregnancy, and that are unlikely to have many eligible pregnant participants. These trials could be used as exemplars, alongside published examples, to support other trials to be more inclusive [7, 17, 27].

The understanding of trials by trialists working on them is one of many factors contributing to inclusion and participation. Different trialists may have different understandings of the same trial, for example, if pregnant people are not explicitly eligible, they may be assumed by some to be ineligible. Trials may be designed in a way that indirectly excludes people during pregnancy, for example, through recruitment only through GPs, while midwives lead care for most pregnant people" to clarify why recruitment only through GPs is a problem.

When given an equitable chance at participation, some pregnant people will choose not to take part in trials; our PPI advisory group told us that they believed that pregnant people should always be “allowed” to make their own choices about participating in ethical research, although they would not all choose to take part in research themselves.

All factors contributing to inclusion and participation need to be considered in combination.

### Limitations

Our PPI contributors, with recent experience of pregnancy, described different experiences, feelings, thoughts, and beliefs. However, we did not assess the diversity of their demographic, social and economic, and health status factors. We will recruit more, and more diverse, contributors to our advisory group.

This study was successful in achieving a high response rate. Given the high response rate and comparable characteristics of responding and non-responding trials, the respondents are likely to be representative of the overall sample. However, we recognise there may have been a response bias. For example, there may have been a lower response rate from potential respondents who felt this was a particularly irrelevant or complex question.

Questions in this survey included examples to aid trialists interpretation of questions. Answers followed a multiple-choice then free-text format to remind trialists of the range of answers that might be relevant. These examples and multiple-choice options may have led trialists to give different or less wide-ranging answers, which we tried to mitigate by using “you might be thinking about” before examples, and providing potential answers “none of these” and “other” ahead of free-text spaces.

As with any survey, responses are subject to individual knowledge, opinions, and feelings. Two contradictory responses were received for one trial but, on reference to the trial protocol, we were able to identify the more accurate response, and more relevant detail that we would have hoped to capture with the survey. However, we were unable to cross-reference all responses against trial documentation, so we do not know how accurate and complete all survey responses are. In-depth interviews with trialists exploring the understanding of trials by trialists working on them, and how this might differ from the design and planned implementation of trials, is a potential for future work.

Many trialists identified at least one way eligibility is documented or implemented, for example in the protocol, or analysis. However, the survey was not successful in eliciting enough information to explore this further.

NIHR HTA-funded trials evaluate interventions already used in the NHS; trials are likely to be phase III or IV. Other, earlier phase trials, are less likely to include people during pregnancy [28]. The NIHR has a strategy for equality, diversity, and inclusion, and provides guidance to support inclusion of under-served groups in research. Trials funded through other organisations may be less likely to include people during pregnancy. Our sample is trials with awards starting between 2022 and 2023; the majority of ongoing trials, and trials on which healthcare policy is based, will be older than this. It is probable that older trials are less likely to include people during pregnancy, meaning that the sample in this survey is likely to be more inclusive of people during pregnancy than many other samples. Exploring inclusion during pregnancy in different types of trials is a potential for future work.

## Conclusions

Exclusion of pregnant people from clinical trials sometimes provides immediate protection to individuals but also subsequently causes harms on a population-level. Despite the NIHR’s clear strategy and guidance to support inclusion of under-served groups, including pregnant people, in clinical trials, a minority of these trials include people during pregnancy. Most trials for which pregnant people are eligible do not explicitly include people during pregnancy or facilitate their inclusion. A small number of trials, different in setting, clinical area, and intervention type, are intentionally designed and conducted in a way that include people during pregnancy. The survey showed that there are clear opportunities to increase the inclusion of pregnant people in clinical trials funded through the NIHR HTA Programme, for example, through explicit and active inclusion, trial design, and provision and use of guidance and support. Future work will include a scoping review searching for facilitators and barriers to inclusion of pregnant people in clinical trials.

## Supplementary Information

Supplementary Material 1.

Supplementary Material 2.

Supplementary Material 3.

## Data Availability

The survey data that support the findings of this study include potentially identifiable data on the respondents; we will not make these data publicly available. However, de-identified data will be made available for research that sets out to achieve aims specified in a methodologically and scientifically sound protocol approved by the University of Oxford Nuffield Department of Primary Care Hosted Research Datasets Committee (PrimDISC). Information regarding how to apply for access to these data should be directed to primdisc@phc.ox.ac.uk.

## Abbreviations

NIHR: National Institute for Health and Care Research
HTA: Health Technology Assessment

## References

[1] Jorgensen SCJ, Miljanic S, Tabbara N, Somanader D, Leung F, De Castro C, et al. Inclusion of pregnant and breastfeeding women in nonobstetrical randomized controlled trials. Am J Obstet Gynecol MFM. 2022;4(6): 100700. 35914736 10.1016/j.ajogmf.2022.100700

[2] Bromley R, Adab N, Bluett-Duncan M, Clayton-Smith J, Christensen J, Edwards K, et al. Monotherapy treatment of epilepsy in pregnancy: congenital malformation outcomes in the child. Cochrane Database Syst Rev. 2023;8(8):Cd010224. 10.1002/14651858.CD010224.pub3 PMC10463554 37647086

[3] Vargesson N. Thalidomide-induced teratogenesis: history and mechanisms. Birth Defects Res C Embryo Today. 2015;105(2):140–56. 26043938 10.1002/bdrc.21096 PMC4737249

[4] Bamigboye AA, Morris J. Oestrogen supplementation, mainly diethylstilbestrol, for preventing miscarriages and other adverse pregnancy outcomes. Cochrane Database Syst Rev. 2003;2003(3):CD004353. 10.1002/14651858.CD004353. PMID: 12918007; PMCID: PMC9039959. 10.1002/14651858.CD004353 PMC9039959 12918007

[5] Söderström M. Why researchers excluded women from their trial populations. Lakartidningen. 2001;98(13):1524–8. 11330148

[6] MBRRACE-UK. Saving lives, improving mothers’ care - lessons learned to inform maternity care from the UK and Ireland confidential enquiries into maternal deaths and morbidity 2023;2019–21. https://www.npeu.ox.ac.uk/mbrrace-uk.

[7] Vousden N, Haynes R, Findlay S, Horby P, Landray M, Chappell L, et al. Facilitating participation in clinical trials during pregnancy. BMJ. 2023;380: e071278. 36746514 10.1136/bmj-2022-071278

[8] Taylor MM, Kobeissi L, Kim C, Amin A, Thorson AE, Bellare NB, et al. Inclusion of pregnant women in COVID-19 treatment trials: a review and global call to action. Lancet Glob Health. 2021;9(3):e366–71. 33340453 10.1016/S2214-109X(20)30484-8 PMC7832459

[9] World Medical Association. World medical association declaration of Helsinki: ethical principles for medical research involving human participants. JAMA. 2025;333(1):71–4. 10.1001/jama.2024.21972. 10.1001/jama.2024.21972 39425955

[10] van der Zande ISE, van der Graaf R, Hooft L, van Delden JJM. Facilitators and barriers to pregnant women’s participation in research: a systematic review. Women Birth. 2018;31(5):350–61. 29373261 10.1016/j.wombi.2017.12.009

[11] The ConcePTION project. The ConcePTION project https://www.imi-conception.eu/2023.

[12] University of Birmingham BHP. Healthy mum, healthy baby, healthy future, the case for UK leadership in the development of safe, effective and accessible medicines for use in pregnancy. 2022. https://www.birminghamhealthpartners.co.uk/healthy-mum-healthy-baby-healthy-future-report/

[13] Medicines and Healthcare products Regulatory Agency. Safer Medicines in Pregnancy and Breastfeeding Information Consortium Information strategy January 2021. Guidance. Medicines and Healthcare products Regulatory Agency. United Kingdom, Medicines and Healthcare products Regulatory Agency. 2021:1-13.

[14] APBI. Maternal health project group (MHPG) APBI; 2023. https://www.abpi.org.uk/partnerships/working-with-patient-organisations/maternal-health-project-group-mhpg/.

[15] NIHR. Equality, diversity and inclusion strategy 2022–2027. NIHR; 2022. https://www.nihr.ac.uk/documents/equality-diversity-and-inclusion-strategy-2022-2027/31295.

[16] National Institute for Health Research. Improving inclusion of under-served groups in clinical research: Guidance from the NIHR-INCLUDE project. 2022. Retrieved 02/07/2025, 2025, from https://www.nihr.ac.uk/documents/improving-inclusion-of-under-served-groups-in-clinical-research-guidance-from-includeproject/25435.

[17] Malhamé I, Hardy E, Cheng MP, Tong SY, Bowen AC. Walking the walk to include pregnant participants in non-obstetric clinical trials: insights from the SNAP Trial. Obstetric Medicine. 2023;16(1):3–4. 37139509 10.1177/1753495X231163351 PMC10150304

[18] van der Zande ISE, van der Graaf R, Browne JL, van Delden JJM. Fair inclusion of pregnant women in clinical research: a systematic review of reported reasons for exclusion. In: Baylis F, Ballantyne A, editors. Clinical research involving pregnant women. Cham: Springer International Publishing; 2016. p. 65–94.

[19] Zur RL. Protected from harm, harmed by protection: ethical consequences of the exclusion of pregnant participants from clinical trials. Research Ethics. 2023;19(4):536–45.

[20] Staniszewska S, Brett J, Simera I, Seers K, Mockford C, Goodlad S, et al. GRIPP2 reporting checklists: tools to improve reporting of patient and public involvement in research. BMJ. 2017;358: j3453. 28768629 10.1136/bmj.j3453 PMC5539518

[21] Anand A, Phillips K, Subramanian A, Lee SI, Wang Z, McCowan R, et al. Prevalence of polypharmacy in pregnancy: a systematic review. BMJ Open. 2023;13(3): e067585. 10.1136/bmjopen-2022-067585 PMC9990613 36878655

[22] Haataja A, Kokki H, Uimari O, Kokki M. Non-obstetric surgery during pregnancy and the effects on maternal and fetal outcomes: a systematic review. Scand J Surg. 2023;112(3):187–205. 37329286 10.1177/14574969231175569

[23] ONS. Conceptions in England and Wales: 2021. ONS; 2023. https://www.ons.gov.uk/peoplepopulationandcommunity/birthsdeathsandmarriages/conceptionandfertilityrates/bulletins/conceptionstatistics/2021.

[24] NIHR. Statement of intent: integrating sex and gender into health and care research. 2023. https://www.nihr.ac.uk/documents/integrating-sex-and-gender-into-health-and-care-research/34906.

[25] Department of Health & Social Care. Women’s health strategy for England. 2022. https://www.gov.uk/government/publications/womens-health-strategy-for-england/womens-health-strategy-for-england.

[26] Trial Forge. Improving trial diversity Trial Forge, 2024. https://www.trialforge.org/improving-trial-diversity/.

[27] Sutton EF, Cain LE, Vallo PM, Redman LM. Strategies for successful recruitment of pregnant patients into clinical trials. Obstet Gynecol. 2017;129(3):554–9. 28178062 10.1097/AOG.0000000000001900 PMC5321786

[28] Ravindran TS, Teerawattananon Y, Tannenbaum C, Vijayasingham L. Making pharmaceutical research and regulation work for women. BMJ. 2020;371: m3808. 33109511 10.1136/bmj.m3808 PMC7587233

