# Supplementary material for: Do pregnant people have opportunities to participate in clinical trials? an exploratory survey of NIHR HTA-funded trialists
Source: Trials. 2025 Jul 4;26:239. doi: 10.1186/s13063-025-08949-w (PMC12232068; doi:10.1186/s13063-025-08949-w)
Supplement: Supplementary file 1 — Supplementary Material 1. [file 13063_2025_8949_MOESM1_ESM.pdf]

Participation in NIHR HTA Programme-funded clinical trials during pregnancy

---

Patient Information Sheet - Page 1

Do pregnant people have opportunities to take part in clinical trials?

General Information

The aim of this project is to assess whether or not National Institute for Health Research (NIHR) Health Technology Assessment (HTA) trials facilitate participation during pregnancy, why, and how.

People are routinely excluded from clinical trials during pregnancy. This means we often don't know with certainty how effective and safe treatments are when someone is pregnant. This is both a practical and an ethical problem. It is difficult for patients and clinicians to make informed choices that result in optimal care. Pregnant people have had treatments which are likely safe and effective withheld or withdrawn, and been harmed (Vousden, Haynes et al. 2023). Principle 13 of the Declaration of Helsinki states that "Groups that are underrepresented in medical research should be provided appropriate access to participation in research"; with regards to pregnant people this is generally not being met (World Medical Association 2013).

Vousden, N., et al. (2023). "Facilitating participation in clinical trials during pregnancy." BMJ 380: e071278.

World Medical Association (2013). Declaration of Helsinki – Ethical Principles for Medical Research Involving Human Subjects.

Many organisations are trying to create high quality evidence and to design individual clinical trials that include people during pregnancy. However, it's difficult to know what change is needed, or being made, when it is not known what current rates of participation are.

This survey will collect the first evidence of rates of eligibility of people during pregnancy in NIHR HTA-funded trials. It will also collect evidence on the rationale and methods to

facilitate (or not) the inclusion of pregnant people in clinical trials funded through the NIHR HTA program. It is part of a larger programme of work under development, with the objective of optimising the participation of pregnant people in clinical trials.

We appreciate your interest in participating in this survey. You have been invited to participate as you are, or have, worked, in any capacity, on a clinical trial funded by the NIHR through their HTA program. Please read through this information before agreeing to participate (if you wish to) by ticking the ‘yes’ box below.

You may ask any questions before deciding to take part by contacting the researcher (details below).

The Principal Researcher is Rebekah Burrow, who is based in the Nuffield Department of Primary Care Health Sciences (NDPCHS) at the University of Oxford. This project is being completed under the supervision of Professor Mike Clarke at Queen’s University Belfast, Dr Lisa Hinton and Professor Rafael Perera, both in NDPCHS at the University of Oxford.

Participants will be asked to respond to a short survey asking about whether, why, and how a specific clinical trial they have worked on facilitates inclusion of pregnant people. This should take about 10 minutes. We appreciate that clinical trials are complex and we are not expecting anyone to have all the answers. Please just answer as best you can! Your knowledge is needed to design future research aimed at facilitating the appropriate inclusion of pregnant people in clinical trials. The answers you provide will be used by the named researchers.

Do I have to take part?

No. Please note that participation is voluntary. If you do decide to take part, you may withdraw at any point for any reason before submitting your answers by pressing the ‘Exit’ button/ closing the browser.

Patient Information Sheet - Page 2

How will my data be used?

The data we will collect that could identify you will be your email address, and a combination of the trial name and your job title.

Your IP address will not be stored. We will take all reasonable measures to ensure that data remain confidential.

The responses you provide will be stored in JISC Online surveys and may be used in academic publications and conference presentations. Data may be stored on backups or server logs beyond the timeframe of this project. All identifiable data will be deleted in September 2030 at the latest (this is the end date of the overall project). Survey data will be stored for a minimum of three years after publication or public release of the work following University of Oxford policy.

If you provide your email address we will use it to contact you with the results of the survey and may use it to request your participation in future closely-related research.

If you wish to update your email address, please email Rebekah Burrow Rebekah.burrow@phc.ox.ac.uk.

Who will have access to my data?

The University of Oxford is the data controller with respect to your personal data and, as such, will determine how your personal data is used in the project. The University will process your personal data for the purpose of the project outlined above. Research is a task that we perform in the public interest. Further information about your rights with respect to your personal data is available from https://compliance.admin.ox.ac.uk/individual-rights.

The data you provide may be shared with the named researchers, and will be accessible by administrators of the survey software, JISC Online surveys.

We would like to use the data in future studies, and to share data with other researchers. Data will have identifying information removed before it is shared with other researchers or results are made public.

The results will be written up for an academic publication. No identifiable data will be published.

Who has reviewed this project?

This project has been reviewed and confirmed not to require ethics clearance by the University of Oxford Research Services.

Who do I contact if I have a concern or I wish to complain?

If you have a concern about any aspect of this project, please speak to Rebekah Burrow Rebekah.burrow@phc.ox.ac.uk or their supervisor Professor Mike Clarke m.clarke@qub.ac.uk, and we will do our best to answer your query. We will acknowledge your concern within 10 working days and give you an indication of how it will be dealt with. If you remain unhappy or wish to make a formal complaint, please contact the Chair of the Research Ethics Committee at the University of Oxford who will seek to resolve the matter as soon as possible:

Medical Sciences Interdivisional Research Ethics Committee; Email: ethics@medsci.ox.ac.uk; Address: Research Services, University of Oxford, Boundary Brook House, Churchill Drive, Headington, Oxford OX3 7GB

1. **Please note that you may only participate in this survey if you are 18 years of age or over.** ✱ *Required*

- ○ I certify that I am 18 years of age or over

2. **If you have read the information above and agree to participate with the understanding that the data (including any personal data) you submit will be processed accordingly, please answer the question below to start.** ✱ *Required*

- ○ Yes, I agree to take part

The clinical trial, and you

3. Which clinical trial are you thinking about when answering this survey? ✱ *Required*

4. What is your job on this trial? Please select the option that matches your job most closely. ✱ *Required*

4.a. If none of these options match your job at all, please tell us what it is here:

Are people eligible to participate in this clinical trial during pregnancy?

From here on the answers you give can lead you to different questions. If you would like to navigate back through the survey to change your answers use the **< Previous** button.

We're hoping to capture a little of the complexity of the reality of clinical trials. Please make use of the free-text boxes as much as you can (but please don't enter any personal or confidential information)!

5. Are people eligible to participate in this clinical trial during pregnancy? ✱ *Required*

- Yes
- No
- I really don't know
- It's complicated...

5.a. If you selected "It's complicated..." can you tell us why? Please make a few notes here to explain.

It's complicated...

6. You answered "It's complicated..." but may still be able to provide some useful answers to the survey. Would you like to try to answer the questions as best you can? (We'd really like you to!) ✱ *Required*

- I would like to try to answer the questions - from the point of view that people are eligible to participate during pregnancy.
- I would like to try to answer the questions - from the point of view that people are NOT eligible to participate during pregnancy.
- Just take me to the end of the survey.

People are eligible for inclusion during pregnancy

What is in place to enable them to participate?

In your answers to the questions below you might be thinking about the following:

Whether inclusion is explicit - **clearly included with no room for confusion or doubt**, or implicit - **suggested though not directly expressed.**

Whether inclusion is purposeful - **having a definite purpose in view**, specific - **clearly defined or identified**, targeted - **directed at a particular group or activity**, or not.

Which stages of the trial impact inclusion - **for example, planning and design, feasibility, consenting, ongoing management, etc.**

Please consider these when making notes to give context to your answer.

For example, the team planning trial A sought funding to include people during pregnancy. Pregnant people are implicitly included in documentation throughout. No measures have been taken to target this specific group for recruitment. The statistical analysis plan does not consider pregnant people as a subgroup.

7. What is in place to enable them to participate? *(tick all that apply)* ✱ *Required*

Please select at least 1 answer(s).

- ☐ There is funding to include them
- ☐ The trials approvals documentation includes them
- ☐ The sponsor's insurer will cover them
- ☐ Trial planning and design documentation includes them
- ☐ Trial management documentation includes them
- ☐ The trial is being conducted in a way that includes them
- ☐ The analysis includes them
- ☐ Regulatory approvals include them
- ☐ None of these
- ☐ Other

8. To add detail and context to your answers above, or if our list prompted you to think of something, please make a few notes here to explain. ✱ *Required*

People are not eligible for inclusion during pregnancy - why?

Why are they not eligible to participate?

For example, trial B doesn't have funding to include people during pregnancy; funding wasn't requested because the sponsor has historically excluded people during pregnancy for safety reasons. In addition, the sponsor considered it highly unlikely that the data generated for pregnant people would be sufficient for a subgroup analysis.

9. Why are they not eligible to participate?*(tick all that apply)* ✱ *Required*

Please select at least 1 answer(s).

- ☐ There is no funding to include them
- ☐ The sponsor's insurer will not cover them
- ☐ Difficulties in obtaining ethical approval
- ☐ Known safety issues
- ☐ Unknown safety issues
- ☐ Statistical issues
- ☐ Difficulties with recruitment or retention
- ☐ Likely no pregnant people will meet the other eligibility criteria
- ☐ None of these
- ☐ Other

10. To add detail and context to your answers above, or if our list prompted you to think of something, please make a few notes here to explain. ✱ *Required*

People are not eligible for inclusion during pregnancy - what is in place to prevent them from participating?

What is in place to prevent them from participating?

In your answers to the questions below you might be thinking about the following:

Whether exclusion is explicit - **clearly excluded with no room for confusion or doubt**, or implicit - **suggested though not directly expressed.**

Whether exclusion is purposeful - **having a definite purpose in view**, specific - **clearly defined or identified**, targeted - **directed at a particular group or activity**, or not.

Which stages of the trial impact exclusion - **for example, planning and design, feasibility, consenting, ongoing management, etc.**

Please consider these when making notes to give context to your answer.

For example, trial B explicitly excluded pregnant people using the eligibility criteria in the protocol and ethical approvals. A screening CRF asks whether a person is pregnant - "yes" leads to not being recruited.

11. What is in place to prevent them from participating?*(tick all that apply)* ✱ *Required*

- ☐ There is no funding to include them
- ☐ The trials approvals documentation excludes them
- ☐ Trial planning and design documentation excludes them
- ☐ The trial is being conducted in a way that excludes them
- ☐ The analysis excludes them
- ☐ Regulatory approvals exclude them
- ☐ None of these
- ☐ Other

12. To add detail and context to your answers above, or if our list prompted you to think of something, please make a few notes here to explain. ✱ *Required*

More information?

Thank you for those answers!

Do you think there are documents related to the trial and our questions that you're able to share?

If so, please email any files to rebekah.burrow@phc.ox.ac.uk

Might there be other people on your team who could provide more information?

If so, please forward the email with the link to this survey to them. We would value hearing from different staff on the same trial.

13. **If you might be open to participating in future closely-related research for this project, or, would like a summary of the results of this survey, please enter your email address.** Data provided cannot be altered or updated once you press “submit”, but you can contact us at rebekah.burrow@phc.ox.ac.uk. *Optional*

Please enter a valid email address.
