# Supplementary material for: Do pregnant people have opportunities to participate in clinical trials? an exploratory survey of NIHR HTA-funded trialists
Source: Trials. 2025 Jul 4;26:239. doi: 10.1186/s13063-025-08949-w (PMC12232068; doi:10.1186/s13063-025-08949-w)
Supplement: Supplementary file 2 — Supplementary Material 2. [file 13063_2025_8949_MOESM2_ESM.docx]

# Supplementary Information

## Additional File 2

These quotes supplement those in the main manuscript.

Respondents volunteered information about why they tried to include people during pregnancy:

**Trial 2** “Pregnancy is not an issue, we didn’t exclude pregnant women”

**Trial 18** “The… trial is a pragmatic… trial.”

**Trial 37** “This is a… trial… unlikely to include pregnant women. However, we felt there was no need to exclude pregnancy as the intervention… fulfills all… requirements so no reason to exclude pregnant women.”

**Trial 55** “There is no reason why pregnant patients could not take part… However, given that the trial is aimed at patients with [condition] very few patients would actually be eligible.

**Trial 69** “There is no specific documentation to enable them to participate but rather the inclusion criteria is any patient who would ordinarily require [intervention]. So, this includes everyone where pregnant or not.”

Some trialists made statements that could be inaccurate:

**Trial 13** “We are not specifically targeting requirement at pregnant people, and are expecting a very low number given that one of our inclusion criteria is 35+.”

**Trial 45** “[Type of intervention] would not be done on females”

**Trial 62** “The… trial is looking at the effects of [intervention] in children (aged 12-18 years)… Pregnancy is not an exclusion criteria but is very unlikely to occur”
